# Quantitative Evaluation of Broadband Photoacoustic Spectroscopy in the Infrared with an Optical Parametric Oscillator

**DOI:** 10.3390/s18113971

**Published:** 2018-11-15

**Authors:** Henry Bruhns, Marcus Wolff, Yannick Saalberg, Klaus Michael Spohr

**Affiliations:** 1 Hochschule für Angewandte Wissenschaften Hamburg, Fakultät Technik und Informatik, Department Maschinenbau und Produktion, Berliner Tor 21, 20099 Hamburg, Germany; henry.bruhns@haw-hamburg.de (H.B.); marcus.wolff@haw-hamburg.de (M.W.); yannick.saalberg@haw-hamburg.de (Y.S.); 2 School of Engineering and Computing, University of the West of Scotland, High Street, Paisley PA1 2BE, UK; 3 Scottish University Physics Alliance (SUPA), University of Glasgow, University Avenue, Glasgow G12 8QQ, UK

**Keywords:** photoacoustic spectroscopy, PAS, hydrocarbons, optical-parametric oscillator, OPO, gas sampling, spectral deconvolution, EUREQA

## Abstract

We evaluate the spectral resolution and the detection thresholds achievable for a photoacoustic spectroscopy (PAS) system in the broadband infrared wavelength region 3270nm≲λ≲3530nm driven by a continuous wave optical parametric oscillator (OPO) with $\bar{P}\;\approx \;1.26\;W$. The absorption spectra, $I_{\mathrm{PAS}}\left(\lambda_{i}\right)$, for diluted propane, ethane and methane test gases at low concentrations (c∼100ppm) were measured for ∼1350 discrete wavelengths $\lambda_{i}$. The $I_{\mathrm{PAS}}\left(\lambda_{i}\right)$ spectra were then compared to the high resolution cross section data, $\sigma_{\mathrm{FTIR}}$, obtained by Fourier Transform Infrared Spectroscopy published in the HITRAN database. Deviations of 7.1(6)% for propane, 8.7(11)% for ethane and 15.0(14)% for methane with regard to the average uncertainty between $I_{\mathrm{PAS}}\left(\lambda_{i}\right)$ and the expected reference values based on $\sigma_{\mathrm{FTIR}}$ were recorded. The characteristic absorption wavelengths $\lambda_{\mathrm{res}}$ can be resolved with an average resolution of $\delta \lambda_{\mathrm{res}}∼0.08\;\mathrm{nm}$. Detection limits range between 7.1 ppb (ethane) to 13.6 ppb (methane). In an additional step, EUREQA, an artificial intelligence (AI) program, was successfully applied to deconvolute simulated PAS spectra of mixed gas samples at low limits of detection. The results justify a further development of PAS technology to support e.g., biomedical research.

## 1. Introduction

Hydrocarbons and other volatile organic compounds (VOCs) are important substances in day-to-day life with regard to e.g., their environmental impact, the exploration of natural gas resources and a manifold of medical applications. With regard to the latter, it has been shown that the exhaled breath of a person includes a complex mixture of thousands of VOCs and precision measurements of their concentrations are very important biomarkers. Among others, their identification can help in the detection of early stage cancers, although a lot of ground work regarding breath collection and data analysis has still to be undertaken [1,2,3,4,5].

Currently, miscellaneous spectroscopic methods in the mid-infrared exist to allow the identification and quantitative measurement of VOCs. Photoacoustic spectroscopy (PAS) is a relatively new technology in that field which only recently has been reviewed and highlighted as a suitable cost-effective, non-destructive and non-invasive spectroscopic method [6,7]. PAS can be performed under atmospheric temperature and pressure conditions with little or no sample preparation on solids, liquids and gases. As such, PAS has the potential to become a versatile standard technique for the detection of VOCs which includes e.g., the aforementioned clinical analysis of exhaled air [8,9,10,11].

PAS facilitates the photoacoustic effect which was independently discovered by A. G. Bell [12] and W. C. Röntgen [13] and describes the transformation of absorbed electromagnetic energy into kinetic energy of the atoms and molecules within the irradiated matter, resulting in thermal expansion. A fast modulation of the triggering radiation supplied e.g., via short flashes of incident laser light will therefore cause periodical fluctuations between thermal expansion and contraction within a selected sample. Under such specific conditions, a sound wave at the modulation frequency is created which can be observed with a sensitive microphone. If the absorbed energy is below the saturation threshold, the amplitude of the sound wave is proportional to the concentration of the molecules in the probe. By measuring the amplitude as function of the wavelength provided by e.g., a tunable laser system, a broadband absorption spectrum can be derived. This allows the identification and quantitative measurement of low concentrations of the specific molecule within the sample if the initial energy of the light source is of adequate magnitude to supply a strong enough signal and the spectral resolution of the PAS system suffices.

To establish PAS technology as a spectroscopic standard, a series of technological advances regarding the reproducibility, handiness and robustness have yet to be achieved [14] and the limit of detection (LOD) needs to be further improved [15]. Choosing a light source with a centre-frequency matching $\lambda_{\mathrm{res}}$ makes frequency tuning expendable and the laser’s repetition rate can be adjusted to the resonance frequency of the photoacoustic cell leading to an optimized single line detection system. Obviously, such a single line system is too limited in resolution to allow a quantitative measure of complex mixtures of gases. Henceforth, the extension and characterization of this promising PAS-based technology into the infrared (IR) broadband regime covering a large number *N* of discrete wavelengths $\lambda_{i}$ with 3270nm≲λ≲3530nm was chosen to be the core rationale behind the presented work.

In detail, the feasibility of an optical parametric oscillator (OPO) as broadband radiation source in conjunction with standard mechanical wheel chopper was investigated by means of qualitative and quantitative evaluation of the obtained PAS spectra for three standard hydrocarbon gases, methane, ethane and propane. Benchmarks included an overall comparison of the measured spectra with the available absorption cross section reference data obtained by Fourier Transform Infrared Spectroscopy (FTIR) for ethane and propane or, for the case of methane, other high precision references depicted in the literature. A numerical evaluation of characteristic absorption lines was undertaken additionally as well as a determination of detection limits and signal-to-noise ratios (SNR). Moreover, we applied a hitherto unprecedented analysis method based on an artificial intelligence evaluation program (EUREQA) for the first time, as we tested whether the qualitative and quantitative parameters obtained with the presented broadband PAS system suffice to deconvolute gas admixtures at ppm level and even below. Finally, the work was also seen as a first step towards the creation of a validated reference database for broadband PAS absorption spectra which could complement the existing data sets for VOC chemicals which are already characterized by high precision IR studies [16].

## 2. Materials and Methods

### 2.1. Experimental Setup

**Figure 1:**
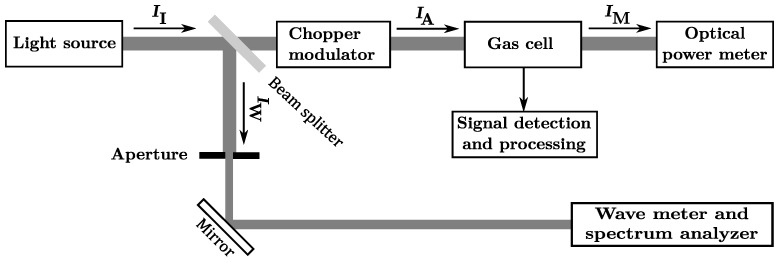
Schematics of the experimental PAS setup.

**Figure 2:**
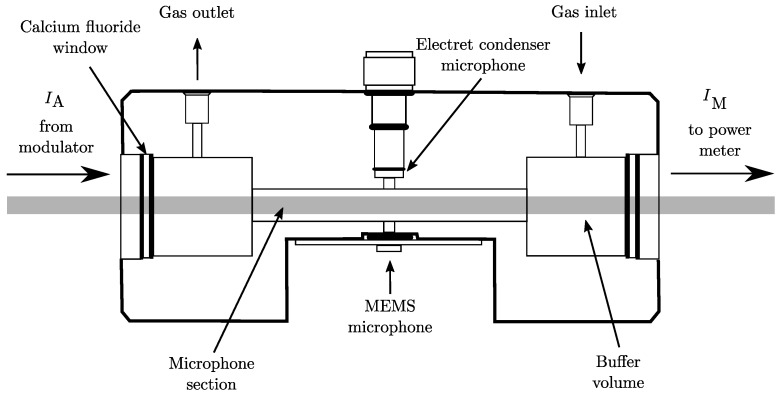
Schematic cross section of the gas cell.

The experimental setup schematically illustrated in Figure 1 is mounted on an optical table. In order to provide an intense light source in the infrared wavelength regime, a continuous wave (cw) OPO is used. The OPO supplies coherent IR radiation in an automatically tunable range between 3200nm and 3700nm with an average step width of $\bar{\delta \lambda_{i}}$ of 0.1865nm. Because the OPO output power *P* changes in a wide range between 0.8 W to 1.6 W, depending on wavelength tuning, *P* has to be measured continuously to allow signal normalization. The original idler beam was split into two by a beam splitter. The strongest beam component with $∼0.93\;\cdot \;I_{I}$ was guided to the chopper modulator which consisted of a motor-driven disc with windows providing a square wave amplitude modulation at a 50% duty cycle. The modulation frequency was aligned to the fundamental longitudinal resonance frequency of a H-type sample gas cell, depicted in Figure 2, of $\bar{f_{\mathrm{mod}}}=2.7\;k\mathrm{Hz}$ with a full-width-half-maximum (FWHM) of 100Hz at room temperature, resulting in a Q-factor of 27. During the measurements, the temperature drift and the frequency variation of $f_{\mathrm{mod}}$ caused a worst case maximum deviation from the cell resonance frequency of ±25Hz. With respect to the frequency response curve of the gas cell, the mismatch could result in a maximum acoustic signal loss of 32%. Due to the fact that the measured spectra should be compared with the shapes of reference spectra and that the measured gas samples are well known, the deduction of absolute quantities of molecules was not in the focus of interest. Therefore, the signal loss due to frequency mismatch could be considered in the comparison with reference spectra. If a quantitative evaluation of unknown gas samples would have been the aim, it would have been appropriate to use a differential chamber cell instead of a simple H-type gas cell, e.g., to allow an in situ calibration with a reference.

The modulated beam had an intensity of $I_{A}∼0.46\;\cdot \;I_{I}$ and was directed to the gas cell which is hermetically closed with two calcium fluoride CaF_2_ windows, transmitting 90% of the incoming light intensity and allowing the constant measurement of the remaining idler wave’s intensity after passage through the cell, $I_{M}$, with a resolution of 3%. The systematic uncertainty is more than twice as high as the total loss of laser power due to absorption in the sample cell ∼1.6% of $I_{A}$ which therefore can be safely neglected in the renormalization of the measured amplitudes. The second, less intense, idler wave component which emerges from the beam splitter $I_{W}=0.07\;\cdot \;I_{0}$, was directed to a combined wavemeter and spectrum analyzer. The wavemeter provided the adjusted wavelength with a nominal accuracy of $1\times 10{}^{-4}\;\mathrm{nm}$ at a resolution of $6\times 10{}^{-4}\;\mathrm{nm}$. In spectrum analyzer mode the FWHM of the idler beam could estimated to be less than 500 pm. More details regarding the setup can be found in Saalberg et al. [11] and Bruhns et al. [17] where an almost identical setup was used.

### 2.2. Measurements

The three lightest straight-chain alkanes and most abundant hydrocarbons, methane (${\mathrm{CH}}_{4}$), ethane ($C_{2}H_{6}$) and propane ($C_{3}H_{8}$) were chosen as test gases for this prima facie study since all of them show strong absorption in the IR regime. All three hydrocarbons were diluted in a nitrogen buffer gas to similar levels of concentration c∼100ppm and were measured sequentially. The spectrum for purified nitrogen gas was determined as well in order to calculate the signal-to-noise ratios. It could be estimated to an average value of $\bar{I_{\mathrm{PAS}}}=0.08$ in arbitrary units (a.u.).

In all measurements, analogue and digital signal detection and processing were applied concurrently for comparison. In the analogue circuit, a condenser microphone was used as detector. The microphone’s output is first preamplified with a voltage gain of 100 before being fed to a digital signal processor (DSP)-based lock-in amplifier. The device is set to a full-scale sensitivity of 500mV and a measurement time constant of 1s. In the digital strand, a highly sensitive microelectromechanical systems (MEMS) microphone was used. The sampling frequency for the signal recording was chosen to be $f_{s}=7.3\;k\mathrm{Hz}$ fulfilling the Nyquist–Shannon theorem. The amplitude of the acoustic signal was calculated in situ by the Goertzel algorithm which uses an efficient evaluation of individual terms of the discrete Fourier transform (DFT) to allow for fast signal processing [18,19,20]. Both methods showed almost identical quantitative results. For simplicity, we only depict the spectra obtained from analog signal processing in the results in Section 3.

Average microphone signal amplitudes, optical power, idler beam wavelength and chopper frequency for sets of 10 measurements were taken for each achievable phase matching condition. A time delay of ∼3 s was allowed for locking. We adjusted a total of N∼1350 discrete wavelengths $\lambda_{i}$ covering the full wavelength region of $3270\;nm≲\lambda_{i}≲3530\;nm$.

**Figure 3:**
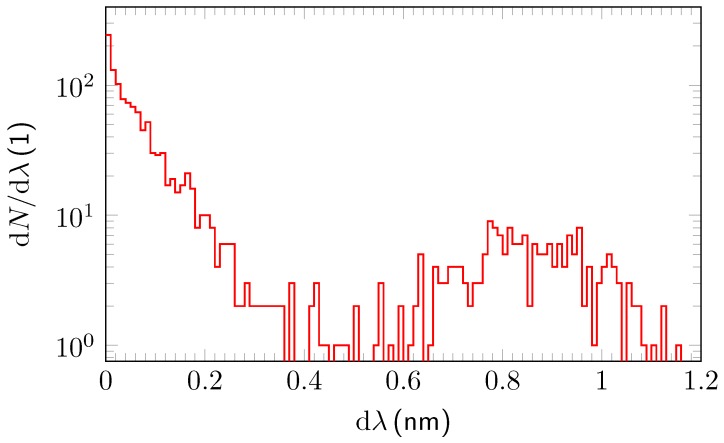
dN/dλ for the propane measurement in bins of 0.1nm. The enhancement of the distribution for 0.6nm≲dλ≲1.1nm is due to non-continuous phase matching at the periodically poled lithium niobate (PPLN) crystal.

The measurement for each of the test gases lasted ∼16 h. The delicate adjusting procedure was heavily influenced by the intrinsic phase matching conditions. Hence, an equidistant spacing $\delta \lambda_{i}=\lambda_{i}-\lambda_{i}-1$ between two successive $\lambda_{i}-1$ and $\lambda_{i}$ was impossible to achieve, resulting in non-continuous spectral tuning steps. Figure 3 depicts the number of phase matching wavelength shifts $\delta \lambda_{i}$ between two consecutive measurements which were sorted in ascending bins of Δλ=0.1nm width to derive the spectra. The non-uniform distribution of the step widths can be clearly deduced from Figure 3. A non-negligible amount of larger step widths occurs for $\delta \lambda_{i}>0.6\;\mathrm{nm}$. On the other hand, if a phase matching condition is met, the output wavelength of the OPO idler beam is very stable. Deviations are mainly affected by temperature changes of the crystal and the OPO cavity. In a separate experiment, the long-term wavelength stability, depending on temperature regulation, was characterized with the result that, during the measurement time interval for 10 data sets taken for every phase matching condition, the wavelength deviation is typically 2pm within the whole tuning range. The maximum long-term control deviation is 60pm within three hours. The transient steepness of the temperature controller leads to a wavelength drift of 0.2pms${}^{-1}$ and the regulating oscillation is 5mHz. An Allan deviation analysis as depicted in detail in e.g., [21] was not undertaken for this experiment as an integration time of 10 s seemed appropriate for the given setup and conditions.

It is also worth pointing out that compared to the high FTIR wavelength resolution, the PAS system has a ∼150 times lower wavelength resolution.

## 3. Results and Interpretation

**Figure 4:**
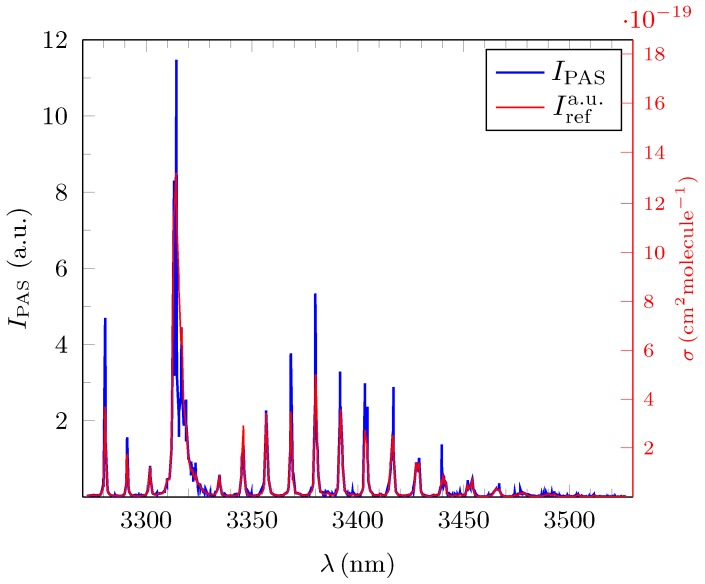
Broadband photoacoustic absorption spectrum (PAS), $I_{\mathrm{PAS}}\left(\lambda_{i}\right)$ (blue) in (a.u.), for methane at c=99.1ppm for N=1350 discrete values of $\lambda_{i}$. The normalized standard reference spectrum $I_{\mathrm{ref}}^{a.u.}\left(\lambda_{i}\right)$ shown in red was calculated from the HITRAN database. The average relative error of $I_{\mathrm{PAS}}\left(\lambda_{i}\right)$ with respect to the reference spectra, $\bar{\delta I_{\mathrm{rel}}}$ is 15.0(14)% (see text for the definition of $\bar{\delta I_{\mathrm{rel}}}$). The red abscissa on the right side refers to the cross section $\sigma_{\mathrm{FTIR}}\left(\lambda_{i}\right)$ and is for guidance only.

**Figure 5:**
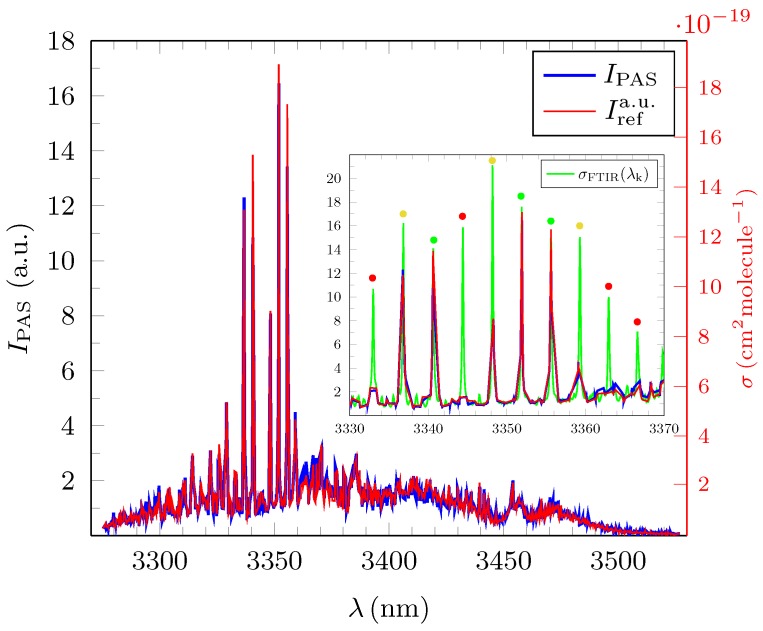
Broadband PAS absorption spectrum $I_{\mathrm{PAS}}\left(\lambda_{i}\right)$ (blue) in (a.u.) for ethane at c=95.5ppm for N=1345 discrete values of $\lambda_{i}$. The normalized standard reference spectrum $I_{\mathrm{ref}}^{a.u.}\left(\lambda_{i}\right)$ (red) was calculated from the HITRAN database. The average relative error, $\bar{\delta I_{\mathrm{rel}}}=$ 8.7(11)%, is small. The inset shows the wavelength region between 3330nm and 3370nm featuring $I_{\mathrm{PAS}}\left(\lambda_{i}\right)$ and $I_{\mathrm{ref}}^{a.u.}\left(\lambda_{i}\right)$ in detail. The selected region is dominated by sharp resonances. The high resolution cross section data set, $\sigma_{\mathrm{FTIR}}\left(\lambda_{k}\right)$, was taken from the HITRAN database and appropriately rescaled (green). Resonances which remained fully unresolved are highlighted with a red circle. Partially resolved resonances are indicated with a yellow circle and accurately resolved ones with a green circle. The cause for the limited resolving capability is discussed in the text.

**Figure 6:**
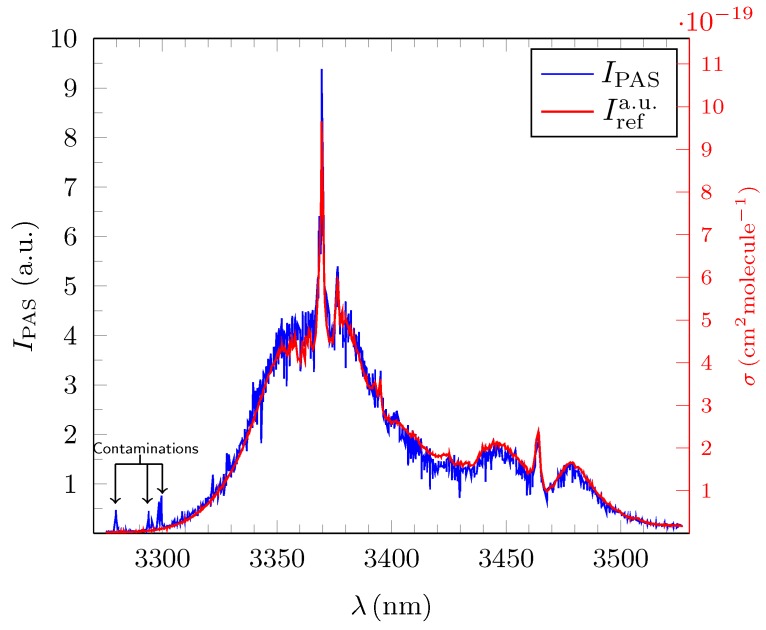
Broadband PAS absorption spectrum $I_{\mathrm{PAS}}\left(\lambda_{i}\right)$ (blue) in arbitrary units (a.u.) for propane at c=99.3ppm for N=1349 discrete values of $\lambda_{i}$. The normalized standard reference spectrum $I_{\mathrm{ref}}^{a.u.}\left(\lambda_{i}\right)$ (red) was calculated from the absorption cross section $\sigma_{\mathrm{FTIR}}\left(\lambda_{k}\right)$ in the HITRAN FTIR database. The average relative error, $\bar{\delta I_{\mathrm{rel}}}$ derived from the 1349 measured wavelengths, after correction for the contaminations, $\lambda_{i}$ is 7.1(6)%, the lowest value of all three test gases.

Figure 4, Figure 5 and Figure 6 show the experimentally obtained PAS absorption signal intensities $I_{\mathrm{PAS}}\left(\lambda_{i}\right)$ in arbitrary units (a.u.) for methane, ethane and propane at ∼100 ppm together with their normalized standard reference spectra $I_{\mathrm{ref}}^{a.u.}\left(\lambda_{i}\right)$ as calculated from the absorption cross sections depicted in HITRAN. The *y*-abscissa on the right (red) of each of these figures represents the standard unit of $1{\mathrm{cm}}^{2}{\mathrm{molecule}}^{-1}$ and relates to the calculated absorption cross sections $\sigma_{\mathrm{ref}}\left(\lambda_{i}\right)$ for each of the three test gases from which the corresponding $I_{\mathrm{ref}}^{a.u.}\left(\lambda_{i}\right)$ were derived. All measured spectra were taken under normal atmospheric temperature and pressure conditions.

### 3.1. Quantitative Evaluation of the Obtained Broadband PAS Spectra for Methane, Ethane and Propane

The measured absorption intensities for all three alkanes at low concentration and relatively low resolution ($\delta \lambda_{i}∼0.18\;\mathrm{nm}$) were compared with the high resolution reference absorption cross sections in the infrared $\sigma_{\mathrm{FTIR}}\left(\lambda_{k}\right)$ as published in the HITRAN (high resolution transmission) molecular absorption database [22]. HITRAN contains a very accurate, self-consistent mixture of direct observations from Fast Fourier transform infrared spectroscopy (FTIR) [23] for a manifold of purified VOCs measured at high concentrations which are complemented by theoretical quantum-mechanical calculations. In the surveyed IR regime $\sigma_{\mathrm{FTIR}}\left(\lambda_{k}\right)$ is given for ∼2.1 × 10${}^{5}$ discrete, equidistant wavelengths $\lambda_{k}$, leading to a high resolution of $\delta \lambda_{k}=0.0012\;\mathrm{nm}$ which is around ∼150 times higher than the resolution in the current PAS measurement. For ethane and propane, $\sigma_{\mathrm{FTIR}}\left(\lambda_{k}\right)$ is published for normal atmospheric temperature and pressure conditions with T∼297.0K and p∼1016hPa in HITRAN. The cross sections are based on the natural isotope abundance, including all isotopologues such as ${}^{13}C^{12}{\mathrm{CH}}_{6}$ for ethane with a natural abundance (NA) of 2.19% and ${}^{13}C^{12}C_{2}H_{8}$ for propane for which NA= 2.12%. A separate measurement of the cross section for ${}^{13}C^{12}{\mathrm{CH}}_{6}$ has only recently been undertaken [24]. The corresponding cross section for methane and its most abundant isotopologue ${}^{13}{\mathrm{CH}}_{4}$ (NA= 1.11%) was calculated from the associated HITRAN lists of absorption lines which included parameters that allowed an evaluation of the air- and self-broadening effects as well as the expected pressure shift.

The reference cross sections $\sigma_{\mathrm{ref}}\left(\lambda_{i}\right)$ for the discrete $\lambda_{i}$ were determined from $\sigma_{\mathrm{FTIR}}\left(\lambda_{k}\right)$ in the relevant wavelength region by a linear fit between the corresponding values for two consecutive wavelengths $\lambda_{k}$ and $\lambda_{k}+1$ in the high resolution spectra which fulfil the condition $\lambda_{k}\leq \lambda_{i}\leq \lambda_{k}+1$ via

$$\sigma_{\mathrm{ref}}\left(\lambda_{i}\right)=\sigma_{\mathrm{FTIR}}\left(\lambda_{k}\right)+\frac{\sigma_{\mathrm{FTIR}}\left(\lambda_{k}+1\right)-\sigma_{\mathrm{FTIR}}\left(\lambda_{k}\right)}{\lambda_{k}+1-\lambda_{k}}\;\cdot \;\left(\lambda_{i}-\lambda_{k}\right)\;.$$

The value of $\sigma_{\mathrm{ref}}\left(\lambda_{i}\right)$ is given in units of $1{\mathrm{cm}}^{2}{\mathrm{molecule}}^{-1}$ at 296K [25] whilst $I_{\mathrm{PAS}}\left(\lambda_{i}\right)$ is given in a.u. for each of the three test gases. In order to compare the measured $I_{\mathrm{PAS}}\left(\lambda_{i}\right)$ and $\sigma_{\mathrm{ref}}\left(\lambda_{i}\right),$ the latter was rescaled into a reference intensity $I_{\mathrm{ref}}^{a.u.}\left(\lambda_{i}\right)$ also given in a.u.,

$$I_{\mathrm{ref}}^{a.u.}\left(\lambda_{i}\right)=\xi_{\mathrm{cor}}\;\cdot \;f_{\mathrm{nor}}\;\cdot \;\sigma_{\mathrm{ref}}\left(\lambda_{i}\right)\;,$$

with $f_{\mathrm{nor}}$ being the normalization factor derived from taking the sum of all measured values of $I_{\mathrm{PAS}}\left(\lambda_{i}\right)$ in a.u. which represents the integrated cross section given by the reference values $\sigma_{\mathrm{ref}}\left(\lambda_{i}\right)$ over the surveyed broadband range. Hence, $f_{\mathrm{nor}}$ could be derived via

$$f_{\mathrm{nor}}=\left(\;\sum_{i=1}^{N}I_{\mathrm{PAS}}\left(\lambda_{i}\right)\;\right)\;\;\cdot \;\;{\left(\;\sum_{i=1}^{N}\;\sigma_{\mathrm{ref}}\left(\lambda_{i}\right)\;\right)}^{-1}\;.$$

The additional parameter $\xi_{\mathrm{cor}}$ is a fitted dimensionless constant for which the total value $\Delta I^{\mathrm{tot}}$,

$$\Delta I^{\mathrm{tot}}\left(\xi_{\mathrm{cor}}\right)=\sum_{i=1}^{N}\;\left|\;I_{\mathrm{PAS}}\left(\lambda_{i}\right)-\xi_{\mathrm{cor}}\;\cdot \;f_{\mathrm{nor}}\;\cdot \;\sigma_{\mathrm{ref}}\left(\lambda_{i}\right)\;\right|\;,$$

of the absolute numerical difference between $I_{\mathrm{PAS}}\left(\lambda_{i}\right)$ and $I_{\mathrm{ref}}^{a.u.}\left(\lambda_{i}\right)$ is minimized and hence their overlap maximized. As such, $\xi_{\mathrm{cor}}$ can be seen as a correction factor. The minimalization process was performed with the background corrected PAS spectra facilitating EUREQA [26], an artificial intelligence powered modelling engine for which we obtained a free academic license courtesy of Nutonian Inc. (Boston, MA, USA). The optimized values for $\xi_{\mathrm{cor}}$ were ∼1 for all three test gases as expected from the obvious similarity of the PAS spectra with the FTIR references (see Table 1). The measured values for $I_{\mathrm{PAS}}\left(\lambda_{i}\right)$ were then compared to $I_{\mathrm{ref}}^{a.u.}\left(\lambda_{i}\right)$ by calculating the average relative error, $\bar{\delta I_{\mathrm{rel}}}$ for all $\lambda_{i}$,

$$\bar{\delta I_{\mathrm{rel}}}=\frac{1}{N}\;\sum_{i=1}^{N}\;\frac{|\;I_{\mathrm{PAS}}\left(\lambda_{i}\right)-I_{\mathrm{ref}}^{a.u.}\left(\lambda_{i}\right)\;|}{I_{\mathrm{ref}}^{a.u.}\left(\lambda_{i}\right)}\;,$$

between the measured distributions $I_{\mathrm{PAS}}\left(\lambda_{i}\right)$ and their corresponding, normalized reference $I_{\mathrm{ref}}^{a.u.}\left(\lambda_{i}\right)$ spectra. The coefficient of determination of the EUREQA fit, $R^{2}$ was later used to help the deconvolution of simulated PAS absorption intensity spectra of mixed gas probes at ppm concentration level (see Section 4). Table 1 summarizes all the deduced crucial parameters for the three measured PAS spectra with *c* representing the concentration of the diluted test gas, $\lambda_{\min}$ the minimal wavelength which was examined and $\lambda_{\max}$ the maximum wavelength examined.

The value of *N* in Table 1 represents the total number of the measured discrete wavelengths $\lambda_{i}$, $I_{\mathrm{PAS}}^{\mathrm{tot}}$ is the total sum of the associated amplitudes in a.u. and a measure of the overall signal strength which is obtainable with the PAS system for any of the three test gases with c∼100ppm. The large uncertainties provided for $\bar{\delta \lambda_{i}}$ are the associated standard deviations of the step size distributions and are large by nature. All values of $\xi_{\mathrm{cor}}$ are very close to 1 emphasizing that the measured spectra $I_{\mathrm{PAS}}\left(\lambda_{i}\right)$ resemble the reference cross section $\sigma_{\mathrm{ref}}\left(\lambda_{i}\right)$ very well, once the initial alignment with $f_{\mathrm{nor}}$ is undertaken. The errors cited for $\bar{\delta I_{\mathrm{rel}}}$ are due to the uncertainties introduced by the background subtraction for the PAS spectra. Some less intensive absorption lines in the wavelength range between 3270 nm to 3350 nm could be assigned to water vapour which was remnant in the gas flow system (see, e.g., Figure 5). A series of additional absorption lines show the presence of more contaminations, e.g., in the wavelength range between 3350 nm and 3380 nm. Due to the incompleteness of the existing databases, it was not possible to identify these small contaminations in due course. However, it needs to be pointed out that these intruders do not substantially influence the rather precise methodology regarding the identification of the three basic hydrocarbons.

The average deviation $\bar{\delta I_{\mathrm{rel}}}$ for all 1351 measured amplitudes $\lambda_{i}$ for propane in the broadband range was only 7.1(6)% underpinning the precision of broadband PAS spectroscopy as can be deduced from Figure 6. The value of $\bar{\delta I_{\mathrm{rel}}}$ for ethane is 8.7(11)% and only slightly higher. Both measurements have the same high $R^{2}$ value, thus further emphasizing the high quality of the PAS measurement.

It is crucial to note that for ethane the measured PAS spectrum does not resolve all of the rather sharp resonances which are clearly visible in the precise $\sigma_{\mathrm{FTIR}}\left(\lambda_{k}\right)$. Some of the resonances are heavily truncated or simply not resolved due to the given distribution of the $\lambda_{i}$ around the resonance peaks amplitude. The inset in Figure 5 shows the wavelength region between 3330nm and 3370nm which is dominated by sharp resonances at specific wavelengths $\lambda_{\mathrm{res}}$ that are resolved accurately with a resolution of $1\times 10{}^{-4}\;\mathrm{nm}$ by FTIR. The appropriately rescaled high resolution $\sigma_{\mathrm{FTIR}}\left(\lambda_{k}\right)$ from HITRAN is depicted in green. Due to the non-continuous varying step sizes $\delta \lambda_{i}$, of the OPO, a total of four of the 10 prominent resonances ($\lambda_{\mathrm{res}}$) in the region situated precisely at 3332.9965 nm, 3344.3997 nm, 3362.9588 nm and 3366.6205 nm remain almost fully unresolved (red circles) in the experiment and another three at 3336.8225 nm, 3348.1816 nm and 3359.3903 nm are only partially resolved (yellow circles) whilst only three resonances at 3340.6194 nm, 3351.0383 nm and 3356.6182 nm (green circles) are accurately resolved. In order to eradicate this artefact in future measurements, $\bar{\delta \lambda_{i}}$ needs to be at least halved for ethane.

Methane has the largest average deviation $\bar{\delta I_{\mathrm{rel}}}$ of 15.0(14)% which is around twice as high as for ethane and propane and coincides with its comparatively low $R^{2}$ value of 0.8260. The reason for the lower quality of the methane PAS spectrum is almost solely of systematic nature since $I_{\mathrm{ref}}^{a.u.}\left(\lambda_{i}\right)$ for methane needed to be be calculated with the help of the line-by-line database in HITRAN as no measured broadband FTIR absorption spectra for methane was published in HITRAN [22]. As such, a discrimination of the weak background features in the measured spectra as in the case for ethane and propane was not possible. In addition, the integral signal amplitude for methane $I_{\mathrm{PAS}}^{\mathrm{tot}}$ shown in Table 1 in the experiment was $<\frac{1}{3}$ of the corresponding values for ethane and propane, thus enhancing the intruding influence of the background signals which were deemed to be of similar magnitude for all three measurements. The quantitative lower quality result in the case of methane should however not distract from the overall very pronounced similarity between the broadband PAS spectra for low concentration levels and the standard FTIR spectra. Figure 4, Figure 5 and Figure 6 and the benchmark parameters supplied in Table 1 clearly evidence the quality of PAS.

### 3.2. Analysis and Quantitative Evaluation of Prominent Absorption Lines

**Figure 7:**
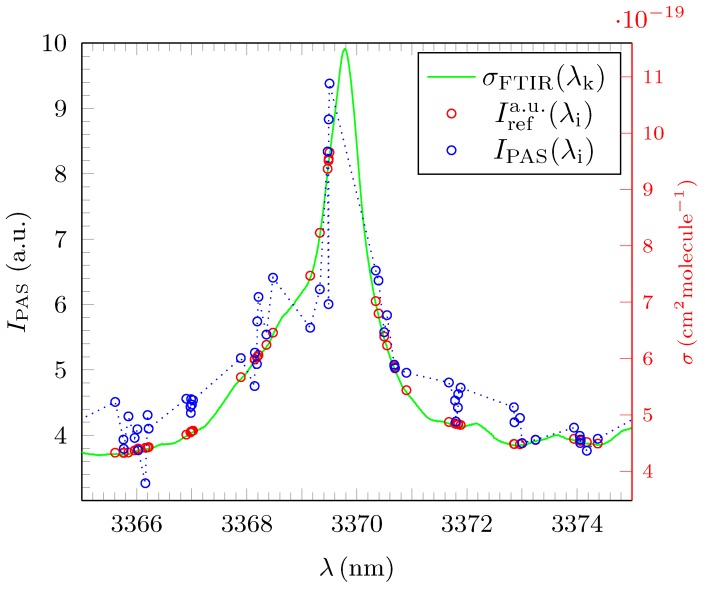
Rescaled absorption cross section $\sigma_{\mathrm{FTIR}}$ of propane for the line at 3369.7628nm at 297K and 1025hPa as published by HITRAN (green). The blue circles show the discrete values for $I_{\mathrm{PAS}}\left(\lambda_{i}\right)$ and the red ones depict the associated reference intensity $I_{\mathrm{ref}}^{a.u}\left(\lambda_{i}\right)$. The high resolution $\sigma_{\mathrm{FTIR}}\left(\lambda_{k}\right)$ is displayed in green colour with its corresponding intensity scale given by the red abscissa on the right.

In a further analysis step, we tested the accuracy of the OPO-driven PAS system with respect to the detection and characterization of distinctive absorption lines which will allow pattern recognition in the quest to identify and to quantify gas admixtures automatically from the obtained photoacoustic spectra with AI programs in the future. Experimentally, these absorption lines exhibit a typical resonance structure which is distinguished by the wavelength $\lambda_{\mathrm{res}}$, the corresponding amplitude $I(\lambda_{\mathrm{res}})$ and the FWHM. The resonance structure is represented by a complex Voigt profile which is a convolution of a Gaussian distribution resulting from Doppler broadening and a Lorentzian distribution caused by pressure broadening [27]. As seen in the previous sub-chapter (see Figure 5), the rather low resolution of PAS caused a series of artefacts concerning the identification of rather sharp resonances, characterized by a small FWHM. Figure 7 which depicts the absorption around the $\lambda_{\mathrm{res}}=3369.7628\;\mathrm{nm}$ absorption line of propane highlights some additional generic problems which need to be considered in the interpretation of benchmark data even in the case of a fully resolved resonance.

The maximum amplitude of the measured photoacoustic signal $I_{\mathrm{PAS}}^{\max}\left(\lambda_{i}^{*}\right)$ appears at a certain wavelength $\lambda_{i}^{*}$ which does not exactly match $\lambda_{\mathrm{res}}$ given by the high resolution $\sigma_{\mathrm{FTIR}}^{\max}\left(\lambda_{k}\right)$ reference spectra. As a result, in the measurement, $I_{\mathrm{PAS}}^{\max}\left(\lambda_{i}^{*}\right)$ for the line at 3369.7628nm only reaches ∼94% of the theoretical highest obtainable value. Moreover, the individual $\lambda_{i}$ are not equally distributed between lower and higher wavelengths around $I(\lambda_{\mathrm{res}})$. Any fit for the position of the amplitude will therefore deviate to a certain degree from the FTIR reference data and systematic discrepancies in the mathematical evaluation of $\lambda_{\mathrm{res}}$, its associated amplitude $I(\lambda_{\mathrm{res}})$ and the associated FWHM may occur. In the example, it can be seen that the actual fit results in a slightly smaller FWHM of the resonance, as the peak seems smaller due to the distribution of the selected wavelengths (see Table 2). Obviously, a too large step size in PAS can also result in artificially enlarged FWHM fit values (see Figure 5), especially for partially resolved, truncated resonances.

To avoid lengthy calculations minimizing the integral expression which characterizes the Voigt profile, a Pseudo–Voigt function $V_{p}\left(\lambda \right)$ was used in the analysis in which the complex integral convolution was replaced by a linear combination of a Lorentzian and Gaussian profiles,

$$L\left(\lambda \right)=\frac{I(\lambda_{\mathrm{res}})}{1+{\left(\frac{\lambda -\lambda_{\mathrm{res}}}{w}\right)}^{2}}\;\;\mathrm{and}\;\;G\left(\lambda \right)=I\left(\lambda_{\mathrm{res}}\right)\;\cdot \;\exp\left[-\ln\left(2\right)\;\cdot \;{\left(\frac{\lambda -\lambda_{\mathrm{res}}}{w}\right)}^{2}\right]\;,$$

$$V_{p}\left(\lambda \right)=\eta \;\cdot \;L\left(\lambda \right)+\left(1-\eta \right)\;\cdot \;G\left(\lambda \right)\;\;\mathrm{for}\;\;0<\eta <1\;.$$

The parameter w in the formula represents the width of the distribution (FWHM=2·w) and the constant η describes the weighting between L(λ) and G(λ). For η=1 the distribution is purely Lorentzian, whilst η=0 represents a pure Gaussian distribution. It is worth pointing out that, in the case of limited experimental resolution, the Gaussian profile also takes precedence over the Lorentzian distribution independent of the influence of Doppler broadening. The minimalization of the absolute difference between $V_{p}\left(\lambda \right)$ and some selected, resolved individual resonance peaks in the background corrected $I_{\mathrm{PAS}}\left(\lambda_{i}\right)$ was undertaken with EUREQA for all three alkanes. EUREQA allowed the simultaneous evaluation of $\lambda_{\mathrm{res}}$, $I(\lambda_{\mathrm{res}})$, the value of the FWHM and the weighting constant η. In the fit procedure, the PAS data were weighted by their amplitudes $I_{\mathrm{PAS}}\left(\lambda_{i}\right)$ to minimize the influence of the low lying noise level. The results are summarized in Table 2.

In Table 2, the expression $\Delta_{\lambda_{\mathrm{res}}}$ represents the relative difference between the experimentally obtained wavelengths for the PAS amplitudes and their corresponding FTIR values. The expression $\Delta_{\mathrm{FWHM}}$ represents the analogon for the FWHM values. Table 2 clearly shows that the positions of the fully resolved resonances are detected properly. The highest relative deviation recorded stands at $\Delta_{\lambda_{\mathrm{res}}}∼4.209\times 10{}^{-5}$ with the average value for $\bar{\Delta_{\lambda_{\mathrm{res}}}}$ being only half that magnitude corresponding to a precision of ∼0.08nm for the mathematical determination of resonant amplitudes from the fit routine. This value is much smaller than the average step size $\bar{\delta \lambda_{i}}$. The uncertainties for all fitted values of $\lambda_{\mathrm{ref}}$ were in all cases negligible and lower than the resolution of the wavemeter of $6\times 10{}^{-4}\;\mathrm{nm}$. Hence, no uncertainty values $\delta \lambda_{\mathrm{res}}$ are explicitly depicted in Table 2 for clarity. Most of the FWHM values show also a good agreement between PAS and the FTIR reference, varying only by $\Delta_{\mathrm{FWHM}}≲20\%$. Some FWHM values are fitted to be lower than their FTIR equivalent which is clearly due to the artefact introduced by the lower resolution of the PAS measurement (see Figure 5 and Figure 7). Some substantially larger FWHM values, especially the one for $\lambda_{\mathrm{res}}=3336.8223\;\mathrm{nm}$ are probably due to the superposition of intruding, unknown absorption lines from an unresolved background of contaminants. The resonances for ethane are almost all pure Lorentzians (η∼1) as expected from the associated FTIR resonances. There corresponding reference FWHM values are ∼0.190 nm which is of the order of the average step size $\delta \lambda_{i}$. This explains why so many resonances in ethane were only partially resolved or even remained completely unresolved in the experiment. We therefore conclude that, if the step size $\delta \lambda_{i}$ compares to the expected FWHM, artefacts of this nature are unavoidable in experimental practise. It is, however, also worth noting, that, if resolved, those sharp ethane resonances could be fitted with the highest values of $R^{2}$, whilst some of the methane resonances showed a rather low value for $R^{2}$ giving further evidence of the systematic deviation in the case of methane. Amplitudes are not included in Table 2 as some of them showed a substantial variation between the values derived for $I_{\mathrm{PAS}}\left(\lambda_{i}\right)$ and those derived from the corresponding reference cross section $\sigma_{\mathrm{FTIR}}$. Variations could be between a few % to factors of three to four if, e.g., the resonance was only partially resolved (see Figure 5). We conclude that line intensities measured with PAS at a resolution which is of the order of the expected line width should only be considered for analysis if a reasonable resolution is achieved and, even then, intensity values should be interpreted with some care.

In summary, Table 2 gives good evidence of the high precision achievable with broadband PAS spectroscopy with respect to the determination of $\lambda_{\mathrm{res}}$ and the corresponding FWHM values which characterize resonant absorption lines. It also highlights the likely appearance of some artefacts which have to be considered in offline analysis, especially if the data obtained is foreseen to inform pattern recognition programs. It is worth pointing out that the influence of these artefacts will scale down substantially with a decreased step size $\delta \lambda_{i}$. A rough estimation would suggest a doubling of *N*, resulting in $\bar{\delta \lambda_{i}}∼0.09\;\mathrm{nm}$ to avoid most of the depicted false fits regarding the FWHM. These artefacts are also present in any other spectroscopic methods which rely on comparable values for $\delta \lambda_{i}$ and are not specifically problems associated with PAS. The data in Table 2 was also used to support the deconvolution calculations as depicted in Section 4.

### 3.3. Estimation of the Signal-to-Noise Ratio (SNR) and the Limit of Detection (LOD)

For an estimation of the signal-to-noise ratio and the limit of detection, the PAS spectra for nitrogen and argon which were used as buffer gases were measured. The average signal level of theses measurements was then folded with the naturally occurring noise floor of the PAS spectra for all the three alkanes. This leads to an overall estimate of the total noise floor of 0.08a.u. for experiments in which nitrogen was used as buffer gas and 0.01 a.u. for those where argon was facilitated. The sensitivity of the detection and the estimation of SNR is furthermore dependent on the minimum observable signal level of $1\times 10{}^{-4}\;mV$ in the analogue signal path and the maximum measured photoacoustic signal $I_{\mathrm{PAS}}^{\max}\left(\lambda_{i}^{*}\right)$ at a certain wavelength $\lambda_{i}^{*}$ which depends on the maximum absorption cross section of the detected test gas and on the optical power provided by the OPO for $\lambda_{i}^{*}$. Since the exact wavelength for any resonance almost certainly will not be exactly matched, as seen in e.g., Figure 7, one can distinct between an experimentally determined lowest limit of detection LOD_exp_ and a corresponding hypothetically equivalent lowest detection limit LOD_hyp_ which would occur if the OPO tuning could exactly be matched to $\sigma_{\mathrm{FTIR}}^{\max}\left(\lambda_{k}\right)$ at maximum OPO output power. The hypothetical value describes the system independently of the distribution of the $\lambda_{i}$ and fluctuations in the output power and is therefore better representing the potentials of the OPO system. The results are summarized in Table 3. A detailed description of the exact procedures involved is given in [28].

In Table 3, $I_{\mathrm{PAS}}^{\max}\left(\lambda_{i}\right)$ represents the maximum measured amplitude in a.u. Uncertainties in these values would be very small and are not listed. The same applies for uncertainties regarding the depicted LOD and SNR values. It can be concluded that the OPO system allows the identification of the measured alkanes down to the low ppb regime.

## 4. Simulation of Deconvolution of Photoacoustic Spectra of Gas Mixtures

Based on the high quality of the obtained spectra for pure alkanes at c∼100ppm, we simulated the expected response of the PAS spectrometer for mixtures of ethane and propane with different relative partial concentrations $c_{\mathrm{par}}^{e}$ and $c_{\mathrm{par}}^{p}$ with $c_{\mathrm{par}}^{e}+c_{\mathrm{par}}^{p}=1000‰$ corresponding to an absolute concentration of 100ppm. This allowed for quantitatively estimating the PAS system’s ability to deconvolute heterogeneous gas probes which will be a crucial benchmark for establishing PAS technology in e.g., the aforementioned medical applications. The deconvolution of the simulated spectra was undertaken with EUREQA and supplemented with measured parameters such as e.g., $\bar{\delta I_{\mathrm{rel}}}$, the uncertainties in determining the position of the resonant lines, $\Delta_{\lambda_{\mathrm{res}}}$ and their corresponding FWHM, $\Delta_{\mathrm{FWHM}}$.

The simulation of the admixtures was based on the existing $\sigma_{\mathrm{FTIR}}$ spectra published in HITRAN which were folded with the quantitative benchmarks obtained for the PAS spectrometer as derived in Section 3. In detail, we selected at first *N* different wavelengths $\lambda_{j}$ in the surveyed region 3270nm≲λ≲3530nm as reference. Simulations were undertaken for N=1350 and N=2700, the latter representing a doubling of the wavelength resolution in the current experiment. This was done by randomly choosing a minimal value for $\lambda_{j=1}∼3530\;nm$ before subsequently generating N−1 additional wavelengths by adding N−1 values of $\delta \lambda_{i}$.

As such, the final simulated $\delta \lambda_{i}$ distribution resembled the resolution in the experiment. The amplitudes $I_{\mathrm{PAS}}\left(\lambda_{j}\right)$ of *N* wavelengths $\lambda_{j}$ were assigned by multiplying the reference values $\sigma_{\mathrm{FTIR}}\left(\lambda_{j}\right)$ with a selected factor so that the measured average relative error, $\bar{\delta I_{\mathrm{rel}}}$ for ethane and propane was identical to the measured values of 8.7% and 7.1%, thus simulating the experimentally achievable resolution for concentrations c∼100ppm for each of the two test gases. Subsequently, the single ethane and propane spectra were weighted and added to simulate a wide variety of relative admixtures from $c_{\mathrm{rel}}=1‰-999‰$ for each gas. It is worth noting that at even the lowest assumed relative concentration of $c_{\mathrm{rel}}=1‰$ in the simulated admixture corresponds to an absolute concentration of c=100ppb, which is still above the experimentally determined LOD (see Table 3). Finally, a random background with an average magnitude of 0.08a.u. as measured was generated.

**Figure 8:**
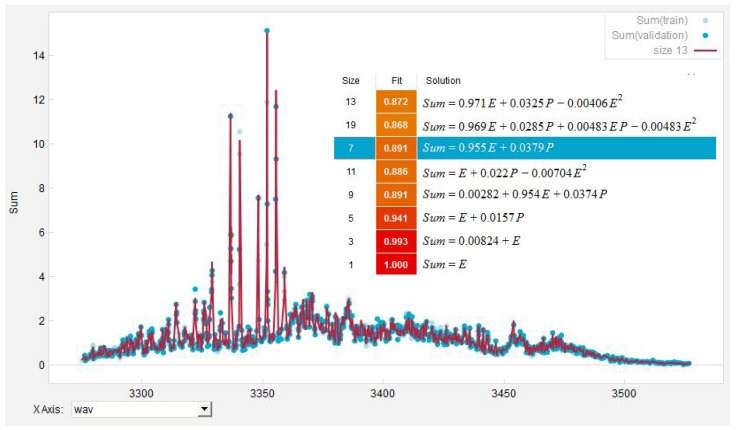
EUREQA analysis of a simulated PAS spectra with 960‰ ethane and 40‰ propane admixture. Selected training points for pattern recognition are annotated with a light blue dot, whilst validation points used to quantify the quality of the fit are indicated with a dark blue dot. Note that the best solution model as found by EUREQA is highlighted in blue.

The simulated spectra were then fitted with the EUREQA program. EUREQA was instructed to search for a numerical combination of the simulated ethane and propane spectra, which leads to the lowest absolute error. To train EUREQA into the recognition of the specific pattern representing the expected PAS spectra of a test gas, the AI program was furthermore informed with the presumed wavelengths of single resonances $\lambda_{\mathrm{res}}$ and the associated uncertainties $\delta_{\lambda_{i}}$. Based on the information provided, EUREQA selects a subset of the presented data to minimize the absolute error and to recognize the expected pattern in case of the resonances. Another independent subset of data is then chosen by EUREQA to evaluate the quality of the fit. Applying this evolutionary data mining concept, EUREQA is able to leverage automated evolutionary algorithms and to create a final accurate predictive model as it will converge to a minimal absolute error. A typical output of the EUREQA program is given in Figure 8.

The quality of the final fit result was classified by the absolute deviation between the concentrations as fitted by EUREQA for ethane and propane $c_{\mathrm{fit}}^{e}$ and $c_{\mathrm{fit}}^{p}$ and the original chosen simulated relative concentrations,

$$\Delta c_{\mathrm{fit}}^{e}=\frac{|\;c_{\mathrm{fit}}^{e}-c_{\mathrm{rel}}^{e}\;|}{c_{\mathrm{rel}}^{e}}\;\;\mathrm{and}\;\;\Delta c_{\mathrm{fit}}^{p}=\frac{|\;c_{\mathrm{fit}}^{p}-c_{\mathrm{rel}}^{p}\;|}{c_{\mathrm{rel}}^{p}}.$$

**Figure 9:**
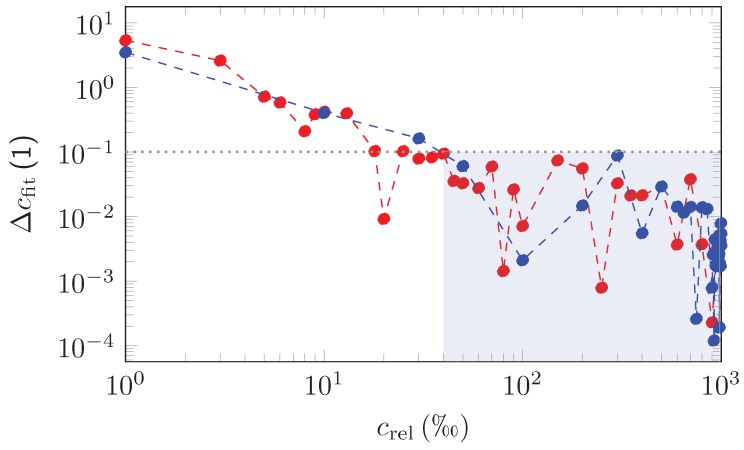
$\Delta c_{\mathrm{fit}}$ as obtained from the comparison between the EUREQA fit and simulated mixed ethane $\Delta c_{\mathrm{fit}}^{e}$ (red dots) and propane $\Delta c_{\mathrm{fit}}^{p}$ (blue dots) spectra based on the current measurements. For relative admixtures with $c_{\mathrm{rel}}\geq 40,$ EUREQA is able to retrieve the concentration with an accuracy better than $10^{-1}=10\%$ (blue area). The dotted lines are depicted to guide the eyes.

Figure 9 shows the results as obtained. It can be deduced from Figure 9 that $\Delta c_{\mathrm{fit}}^{e}$ and $\Delta c_{\mathrm{fit}}^{p}$ behave in a very similar way with the relative deviation declining rapidly in general for increasingly higher values of relative concentrations. For relative concentrations $c_{\mathrm{rel}}<5‰,$ the fitted concentrations $c_{\mathrm{fit}}^{e}$ and $c_{\mathrm{fit}}^{p}$ are off by factors of 2–6 compared to the simulated concentrations, but still the deviation remains below a full order of magnitude. Relative deviations between $10^{-1}$–1 are to be expected for $5‰<c_{\mathrm{rel}}<40‰$ and, for $c_{\mathrm{rel}}>40‰,$ EUREQA is able to retrieve the true values of the concentration with an accuracy better than $10^{-1}=10\%$ for ethane and propane, which has to be seen as a good result.

## 5. Conclusions

We presented an exhaustive evaluation of OPO-driven infrared photoacoustic broadband spectroscopy covering the spectral range between 3270 nm to 3530 nm with an average resolution (step size) of $\bar{\delta \lambda_{i}}=0.18\;\mathrm{nm}$ for propane, ethane and methane at concentrations of c∼100ppm. Figure 4, Figure 5 and Figure 6 clearly demonstrate that, with the given parameters, absorption spectra of alkenes can be derived with sufficient quality. As a suitable quantitative benchmark, we introduced the average relative error per channel, $\bar{\delta I_{\mathrm{rel}}}$ between the measured spectral amplitudes and the corresponding normalized intensities from the FTIR spectra as depicted in the HITRAN database. Values for $\bar{\delta I_{\mathrm{rel}}}$ ranged between 7.1(6)% (propane)–15.0(14)% (methane). This result is quite remarkable as the FTIR standard has a ∼150 fold increased resolution compared to the average step width in the experimental PAS spectra of ∼0.18 nm. In a further step, the average precision with which the position of the amplitudes of the sharp resonances could be resolved was found to be 0.08 nm, which is less than $\delta \lambda_{i}$. If fully resolved by a Pseudo–Voigt fit, the measured FWHM could be determined correctly and compared well with the FTIR standard. However, as due to technical limitations, the step sizes varied a lot. This lead to a high standard deviation regarding the average step width for the wavelength and a series of artefacts occurred in the spectraMost noticeable were the too small values for the FWHM of some partially resolved resonances. Moreover, some resonances could not be resolved at all and therefore the amplitude of the single resonance was deemed not to be a desirable benchmark. From estimates, we concluded that the occurrence of these artefacts can be strongly suppressed by reducing $\delta \lambda_{i}$ by a factor of two or more in future measurements. Experimentally determined detection limits ranged from 7.1ppb–13.6 ppb and signal-to-noise ratios from 117.3‰–205.7. Informed by this gamut of parameters, we simulated the deconvolution of different admixtures of ethane and propane with the help of EUREQA, an AI program. We found that, even if the less prevalent gas has a concentration of c∼4ppm corresponding to only 40‰ in the mixed gas, its total abundance could be still be determined with an accuracy of ≲10%.

We hope this work introduces simple benchmarks that allow a quantification of the quality of PAS spectra in the near future. Moreover, we suggest further work in the measurement and simulation of gas admixtures with PAS and their analysis with the help of an AI program such as EUREQA. Our work demonstrates the suitability of a modern OPO-driven laser system to become a reference tool in photoacoustic spectroscopy.

## Figures and Tables

**Table 1:** Parameters of the measured broadband $I_{\mathrm{PAS}}$ spectra for methane, ethane and propane and related quantitative benchmark parameters as derived from EUREQA.

	Measurement						EUREQA-Fit		
	c/ppm	$\lambda_{\min}$/nm	$\lambda_{\max}$/nm	N	$\bar{\delta \lambda_{i}}$/nm	$I_{\mathrm{PAS}}^{\mathrm{tot}}$/a.u.	$\bar{\delta I_{\mathrm{rel}}}$/%	$\xi_{\mathrm{cor}}$	$R^{2}$
Methane	99.1	3272.0361	3526.8055	1350	0.1887(3025)	464.1	15.0(14)	1.0288	0.8260
Ethane	95.5	3275.2941	3526.8729	1345	0.1870(2926)	1593.9	8.7(11)	0.9959	0.9759
Propane	99.5	3275.3858	3526.9183	1351	0.1865(2931)	2170.7	7.1(6)	0.9996	0.9760

**Table 2:** Comparison of the position and FWHM of selected resonance lines in diluted methane, ethane and propane test gases as obtained by PAS and the corresponding FTIR reference values.

	$\lambda_{\mathrm{res}}$/nm		$\Delta_{\lambda_{\mathrm{res}}}$/$10^{-5}$	FWHM/nm		$\Delta_{\mathrm{FWHM}}$/%	EUREQA-Fit	
	PAS	FTIR		PAS	FTIR		η	$R^{2}$
Methane	3280.5219	3280.6543	−4.036	0.568	0.641	−11.50	0.5313	0.9354
	3291.1426	3291.0667	2.306	0.599	0.738	−18.84	0.6196	0.9976
	3368.6480	3368.5638	2.500	0.745	0.996	−25.16	0.6205	0.9231
	3391.9170	3392.0495	−3.906	1.636	1.376	18.85	0.1047	0.7012
	3428.1770	3428.1805	−0.102	2.321	2.361	−1.69	0.0005	0.8692
	3465.8520	3465.7252	3.659	3.317	2.823	17.49	0.2794	0.6394
Ethane	3336.7143	3336.8223	−3.237	0.275	0.178	54.46	1.0000	0.9416
	3340.5772	3340.6186	−1.389	0.158	0.197	−19.83	0.9999	0.9981
	3348.2759	3348.1813	2.825	0.176	0.181	−3.14	0.9995	0.9990
	3351.9117	3351.8977	0.418	0.139	0.179	−22.06	0.5437	0.9954
	3355.6083	3355.9151	−0.203	0.231	0.198	18.17	0.9994	0.9999
Propane	3369.8481	3369.7503	2.902	0.653	0.792	−17.62	0.5056	0.8014
	3463.6431	3463.7889	−4.209	2.268	2.072	9.44	0.5533	0.8147

**Table 3:** Experimental and hypothetical detection limits (LOD) and signal-to-noise (SNR) ratios of the OPO driven broadband PAS system.

		Experiment		Hypothetical	
	$I_{\mathrm{PAS}}^{\max}\left(\lambda_{i^{*}}\right)$/a.u.	${\mathrm{LOD}}_{\exp}$/ppb	${\mathrm{SNR}}_{\exp}$	${\mathrm{LOD}}_{\mathrm{hyp}}$/ppb	${\mathrm{SNR}}_{\mathrm{hyp}}$
Methane	11.4747	13.6	143.4	3.0	227.9
Ethane	16.4530	7.1	205.7	2.4	270.3
Propane	9.3811	13.2	117.3	4.9	137.3
Nitrogen	0.0800		1.0		

## Abbreviations

The following abbreviations are used in this manuscript

AI	Artificial Intelligence
FTIR	Fast Fourier transformation in the infrared
FWHM	Full-width-half-maximum
IR	Infrared
LOD	Limit of detection
MEMS	Microelectromechanical systems microphone
NA	Natural abundance
OPO	Optical-parametric oscillator
PAS	Photoacoustic spectroscopy
PPLN	Periodically poled lithium niobate
SNR	Signal-to-noise ratio
